# Determination of Vancomycin in Human Serum by Cyclodextrin-Micellar Electrokinetic Capillary Chromatography (CD-MEKC) and Application for PDAP Patients

**DOI:** 10.3390/molecules22040538

**Published:** 2017-03-28

**Authors:** Jiajing Wang, Yuqing Cao, Shengyuan Wu, Shuowen Wang, Xin Zhao, Tingting Zhou, Yuefen Lou, Guorong Fan

**Affiliations:** 1 Department of Clinical Pharmacy, Shanghai General Hospital, School of Medicine, Shanghai Jiao Tong University, No. 100 Haining Road, Shanghai 200080, China; wangjiajing77@163.com (J.W.); wangshuowensy@163.com (S.W.); 2 Shanghai Key Laboratory for Pharmaceutical Metabolite Research, School of Pharmacy, Second Military Medical University, Shanghai 200433, China; caoyuqing1109@163.com (Y.C.); tingting_zoo@163.com (T.Z.); 3 Laboratory of Drug Metabolism & Pharmacokinetics, School of Medicine, Tongji University, No. 1239 Siping Road, Shanghai 200092, China; wu-shengyuan@tongji.edu.cn; 4 Department of Pharmaceutical Analysis, School of Pharmacy, China Pharmaceutical University, No. 24 Tong Jia Xiang, Nanjing 210009, China; yoyo0132@163.com; 5 Department of Pharmacy, Shanghai Fourth People’s Hospital, Shanghai 200081, China

**Keywords:** vancomycin, cyclodextrin-micellar electrokinetic capillary chromatography, peritoneal dialysis-associated peritonitis

## Abstract

A simple and sensitive cyclodextrin-micellar electrokinetic capillary chromatography (CD-MEKC) method with UV detection was developed and validated for the determination of vancomycin (VCM) in serum. The separation was achieved in 14 min at 25 °C with a fused-silica capillary column of 40.2 cm × 75 μm i.d. (effective length 30.2 cm) and a run buffer containing 25 mM borate buffer with 50 mM sodium dodecylsulfonate (SDS) (pH 9.5) and 2% sulfobutyl-β-cyclodextrin (sulfobutyl-β-CD). Under optimal conditions for biological samples, good separations with high efficiency and short analysis time were achieved. Several parameters affecting the drug separation from biological matrices were studied, including buffer types, concentrations, and pHs. The methods were validated over the range of 0.9998–99.98 µg/mL. Calibration curves of VCM also showed good linearity (*r*^2^ > 0.999). Intra- and interday precisions (relative standard deviation, RSD) were less than 5.80% and 7.38%, and lower limit of quantification (LLOQ) were lower than 1.0 μg/mL. The mean recoveries ranged between 84.03% and 91.69%. The method was successfully applied for monitoring VCM concentrations in serum of patients with peritoneal dialysis-associated peritonitis (PDAP). The assay should be applicable to pharmacokinetic studies and routine therapeutic drug monitoring of this drug in serum.

## 1. Introduction

**Figure 1:**
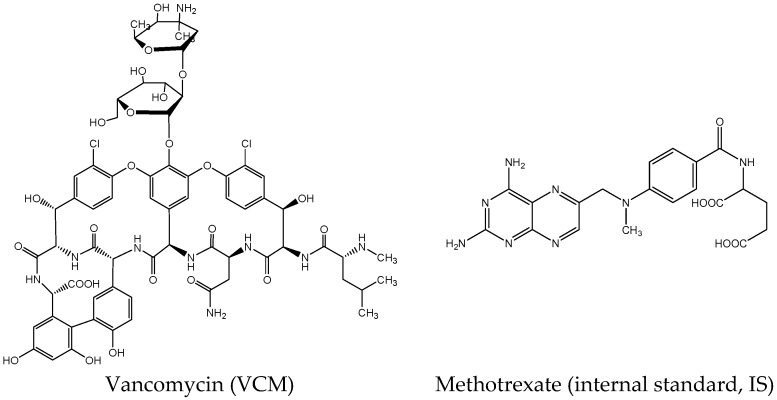
Chemical structures of vancomycin (VCM) and methotrexate (IS).

Peritoneal dialysis-associated peritonitis (PDAP) is a serious complication in peritoneal dialysis patients. It is important to reduce the risk of peritoneal dialysis-associated infection. Vancomycin (VCM) is a glycopeptide antibiotic isolated from *Streptomyces orientalis*, and is widely used in the prophylaxis and therapy against infections by Gram-positive bacteria, including methicillin-staphylococcus aureus (MRSA) [1,2]. The International Society for Peritoneal Dialysis (ISPD) recommended VCM for the treatment of PDAP caused by infection with MRSA and other Gram-positive bacteria [3]. Consequently, serum-level monitoring of VCM is necessary to ensure adequate therapeutic concentrations while avoiding toxic accumulations. The chemical structure of VCM is shown in Figure 1. Several methods for the determination of VCM in various biological fluids have been reported, such as radioimmunoassay (RIA) [4,5], fluorescence polarization immunoassay (FPIA) [6,7], enzyme-multiplied immunoassay (EMIT) [8], high performance liquid chromatography (HPLC), and liquid chromatography coupled with mass spectrometry (LC/MS) [9,10,11]. Among these techniques, FPIA is the recommended method by clinical laboratories because of its convenience and high-throughput capability. However, overestimation was considered by accumulation of cross-reacting degradation products [12,13]. HPLC is also widely used for its sensitivity, specificity, and precision, although it is not the simplest or least time-consuming [12,13,14]. More recently, LC-MS has been developed for the analysis of VCM. There is thus no doubt that owing to their high sensitivity and specificity, they offer optimum methodology for the quantification of VCM. However, they may not be feasible because of the expensive technology required for their implementation [15,16]. Capillary electrophoresis (CE) is a miniaturized technique that offers some advantages with respect to chromatography techniques, such as its high efficiency, resolution, and low reagents organic solvents consumptions, and with the consequent decrease of waste generation, environmental pollution and cost [17,18]. Several CE methods have been developed to determine VCM for assay, related impurities, and concentrations in biological matrices [14,19,20,21,22,23]. As far as we know, only a few have been published for determining VCM concentrations in patients’ plasma or serum for therapeutic drug monitoring (TDM). To determine VCM concentration in patients’ serum, a micellar electrokinetic capillary chromatography (MEKC) method with direct sample injection was reported by Kitahashi et al. [20]. In order to achieve serum proteins’ solubilization and co-elution with micelle, 100 mM SDS was used in this study. Considering the experiment in practice, unexpected current interruption was more likely to happen by introducing high SDS concentration. Yang et al. [21] published an MEKC method for simultaneous analysis of cefepime and VCM in two meningitis patients’ plasma and in cerebrospinal fluid. Eighteen percent methanol was used in background electrolyte (BGE) for better resolution and capacity factor. Based on our experimental experience, the addition of methanol will also easily lead to current interruption. Moreover, 300 mM SDS was used for separation. In our paper, a cyclodextrin (CD)-MEKC method based on protein precipitation using 25 mM borate buffer, 50 mM SDS, and 2% sulfobutyl-β-CD (pH 9.50) was successfully developed for the monitoring of VCM through serum concentrations in PDAP patients. All these samples were simultaneously detected by HPLC and CE. Concentrations in the range 8.53–35.22 μg/mL were obtained by CE and 9.30–33.25 μg/mL were obtained by HPLC. Clinicians can adjust dosage according to serum concentrations according to ISPD. These results indicated that this CE method can be an alternative method for VCM-TDM of PDAP patients.

## 2. Results and Discussion

### 2.1. Method Development

#### 2.1.1. Optimization of Buffer Type and the Concentration

At the beginning of this experiment, three different types of buffers (phosphoric acid-sodium phosphate, Tris-phosphoric acid-sodium phosphate-SDS, and borate-SDS) were studied. The peak shapes of analytes were good when we used Na_2_B_4_O_7_-SDS (pH 9.5). Buffer concentration was the second investigation parameter in our study. The current increased as the buffer concentration increased, which led to more joule heating and longer analysis time. However, lower sensitivity was observed as the ionic strength decreased. On the other hand, the negative charge of the anionic surfactant SDS may increase the sensitivity, owing to the modified conductivity of background electrolyte (BGE) and the EOF (electroosmotic flow) velocity. The effects of SDS at a concentration 25 mM Na_2_B_4_O_7_-20 mM SDS (pH 9.5), 25 mM Na_2_B_4_O_7_-50 mM SDS (pH 9.5), and 50 mM Na_2_B_4_O_7_-50 mM SDS (pH 9.5) were investigated. Better peak shapes and proper migration times were observed by using 25 mM Na_2_B_4_O_7_-50 mM SDS (pH 9.5), and thus were chosen.

#### 2.1.2. Effect of pH

The pH value of BGE is an important factor in CE. VCM is a glycopeptide antibiotic with quite a complex structure. It contains six functional groups which participate in acid-base equilibria [24]. Namely, its net charge in solution strongly depends on the solution pH. The p*K*_a_ of 2.18 contributes from the carboxyl group. Protonations of amino (primary and secondary amino) and three phenolate groups take place between pH 13 and 5 in multiply overlapping stages. As the literature shows, VCM p*K*_a_ values are 2.18, 7.75, 8.89, 9.59, 10.40 and 12.00, and the pI value is about 7.10 [25]. Owing to the positive charge of VCM and internal standard (IS) (methotrexate, MTX) under acidic BGE, the interaction of the analytes with the wall of fused-silica capillary was occurred. As a result, longer migration time, less repeatable and serious peak tailing were observed. As a result, the effect of buffer pH (8.5, 9.0, 9.5, 10.0 and 10.5) of 25 mM Na_2_B_4_O_7_ with 50 mM SDS was investigated. The EOF increased obviously as pH increased, which led to the decreasing of migration time. Current interruption occurred when pH was 10.0 and 10.5. Considering proper migration time and good resolution, pH 9.5 was chosen for the further investigation.

MTX is an important chemotherapeutic agent. In our further study, we hope its serum-concentration can also be detected for reducing incidences of toxicity in cancer patients by using the same method as in this paper.

#### 2.1.3. Effect of β-CDs

**Figure 2:**
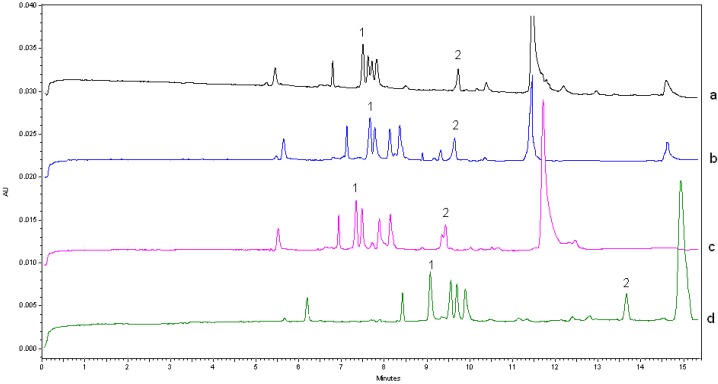
Electropherograms of serums spiked VCM and IS with different types of β-cyclodextrins (β-CDs) in the background electrolyte (BGE): (**a**) 0.5% Methyl-β-CD; (**b**) 2.0% Methyl-β-CD; (**c**) 2.0% hydroxypropyl-β-CD; (**d**) 2.0% Sulfobutyl-β-CD. Experimental conditions: running buffer: 25 mM Na_2_B_4_O_7_ with 50 mM SDS (pH 9.5); total uncoated capillary length: 40.2 cm × 75 μm i.d., effective length: 30.2 cm; applied voltage: 15 kV (+) → (−); column temperature: 25 °C; detection wavelength: 210 nm; hydrodynamic injection: 0.5 psi × 5 s.

The serum spiked with VCM and IS was studied by 25 mM Na_2_B_4_O_7_ with 50 mM SDS (pH 9.5). Interference occurred in endogenous substance-VCM. Cyclodextrins (CDs) are oligosaccharides with truncated cylindrical molecular shapes. Their outside surfaces are hydrophilic, while their cavities are hydrophobic. They are often used for analogue separation by including compounds which fit their cavities by hydrophobic interaction. Methyl-β-cyclodextrin (Methyl-β-CD), hydroxypropyl-β-cyclodextrin (hydroxypropyl-β-CD), and sulfobutyl-β-cyclodextrin (Sulfobutyl-β-CD) were investigated in our study. As shown in Figure 2, the separation of both endogenous substance-VCM and endogenous substance-IS was not obviously improved when methyl-β-CD and hydroxypropyl-β-CD were used. By adding 2% sulfobutyl-β-CD, better baseline separations were obtained.

#### 2.1.4. Effect of Voltage

Different separation voltages were also tested, such as 10 kV, 15 kV and 20 kV. Too large a separation voltage could cause certain current problems, including high joule heating and low column efficiency. A high column efficiency and short separation time could be obtained when 15 kV (+) → (−) was chosen in the experiments.

### 2.2. Method Validation

#### 2.2.1. Specificity

**Figure 3:**
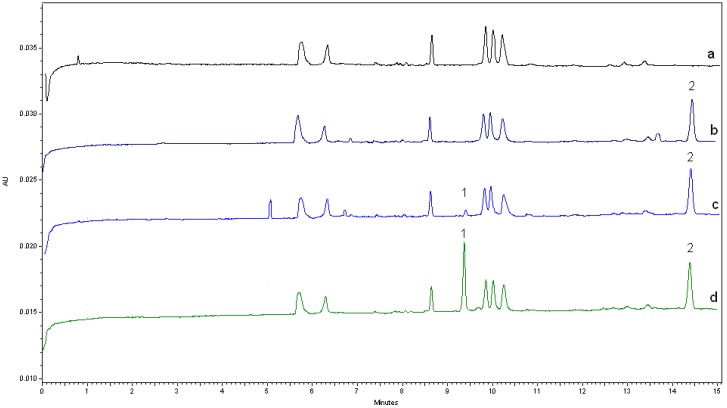
Typical chromatograms of blank plasma: (**a**) blank plasma spiked with IS; (**b**) blank plasma spiked with 1.0 µg/mL (lower limit of quantification, LLOQ) VCM and IS; (**c**) test plasma from peritoneal dialysis-associated peritonitis (PDAP) patient spiked with IS; (**d**) Peak 1: VCM; peak 2: IS. Experimental conditions: running buffer: 25 mM Na_2_B_4_O_7_ with 50 mM SDS (pH 9.5) and 2% sulfobutyl-β-CD. Other conditions are the same as indicated in Figure 2.

The specificity of the method was investigated by blank serum, blank serum spiked with IS, blank serum spiked with VCM (lower limit of quantification, LLOQ) and IS, and a PDAP patient’s serum sample to discriminate the analyte from all potentially interfering substance (Figure 3). Peak purity was evaluated by means of the P/ACE System MDQ Software. The total peak purity values of VCM and IS was 1.0000. There were no interfering peaks from the endogenous substances observed at migration times of about 9.3 min for VCM and about 14.3 min for the IS, which were analyzed under the same optimized condition.

#### 2.2.2. Linearity of Calibration Curves and Lower Limit of Quantification (LLOQ)

The linearity of the CD-MEKC method was evaluated by analysis of seven concentration samples of VCM (1, 2, 5, 10, 20, 50, 100 µg/mL); each sample had a concentration of 10 µg/mL of IS. The calibration equation was *y* = 0.1006*x* − 0.0674, *r*^2^ = 0.9997. In this equation, *y* represents the peak area ratios of the analyte to the IS, and *x* represents the serum concentration of analyte. The calibration curve was achieved with a 1/*x* weighing factor. The LLOQ of VCM in human serum was found to be 1 µg/mL with accuracy of 117.07% and precision of 2.00% (Table 1).

#### 2.2.3. Accuracy, Precision, and Extraction Recovery

Five replicate samples of quality control (QC) at three levels (low, medium, and high concentrations, 3, 15, 80 µg/mL) were analyzed in three separate runs. Precision was determined by calculating the coefficient of variation for within- and between-run replicates. Accuracy was assessed by calculating the percentage deviation from the spiked concentration. Table 1 showed that the within-day variances were lower than 5.80% and all between-day variances were below 7.38%. Recovery from human serum samples was evaluated in VCM for three levels, the response for each concentration being compared with that from the corresponding standard solution. The extraction recovery values are shown in Table 2.

#### 2.2.4. Stability

Analyte stability determinations comprised short-term temperature stability (bench-top stability), long-term stability (30-day stability), autosampler stability, and freeze-thaw cycles stability, which were evaluated by analyzing three QC levels in VCM. The peak areas obtained from both stability tests were compared with the peak areas obtained with freshly prepared samples. The mean values and standard deviations of the ratios between the concentrations found and initial concentration were used for stability evaluation. Table 3 shows that VCM in the spiked serum samples was found to be stable in the experimental conditions assayed.

## 3. Experimental Section

### 3.1. Chemicals and Reagents

VCM and internal standard (methotrexate, MTX) were purchased from Dalian Meilun Biotech Co., Ltd. (Dalian, China). All reagents used were of analytical-reagent grade. Acetonitrile (gradient grade for LC) was supplied by Merck (Darmstadt, Germany). NaOH, sodium tetraborate (Na_2_B_4_O_7_), SDS, sulfobutyl-β-CD, hydroxypropyl-β-CD, and sulfobutyl-β-CD were acquired from China Medicine (Group) Shanghai Chemical Reagent Corporation (Shanghai, China). Deionized water (18 MΩ/cm) was generated in-house using a Milli-Q System from Millipore (Bedford, MA, USA). All solutions were degassed by ultrasonication (KQ-50DE, Kunshan Ultrasonic Instrument Co., Ltd., Kunshan, China). Human control serum was obtained from Shanghai Blood Center (Shanghai, China). The SiroccoTM 96-well plates were purchased from Waters Corporation (Milford, CT, USA).

### 3.2. CE Instrumentation

A Beckman P/ACE MDQ system (Fullerton, CA, USA) equipped with a photo DAD (190–600 nm) and a liquid-cooling device were used. Analysis was carried out in an uncoated fused-silica capillary (Yongnian Photoconductive Fiber Factory, Hebei, China) of 40.2 cm (effective length 30.2 cm) with 75 μm i.d. The temperature of the separation was controlled at 25 °C. The detector was set at 210 nm (cathode at the detection side). The Beckman 32 Karat Software System (version 7.0, Beckman, Brescia, CA, USA) was used for data processing.

### 3.3. Capillary Electrophoretic Conditions

The BGE used in this study was 25 mM borate buffer with 50 mM SDS and 2% sulfobutyl-β-CD (pH 9.50). It was prepared by weighting appropriate amounts of Na_2_B_4_O_7_∙10H_2_O, SDS, and sulfobutyl-β-CD, followed by thoroughly mixing and dissolving with 100 mL deionized water. NaOH (1 mol/L) was used for adjusting pH to 9.50. BGE solutions were prepared freshly every day and filtered through a 0.45 μm hydrophilic cellulose membrane filter prior to use. The separation voltage was 15 kV (+) → (−). Hydrodynamic injection mode was performed at a pressure of 0.5 psi for 5 s. The capillary was conditioned by rinsing with 0.1 mol/L NaOH (1 min) followed by BGE (5 min) sequentially between the runs in order to maintain an active and reproducible inner surface.

### 3.4. Preparation of Stock Solutions, Calibration Samples, and Quality Control Samples

Standard stock solution (1 mg/mL) of VCM and the IS were prepared in methanol. Working standard solutions (ranging from 10 to 500 µg/mL) were prepared daily by diluting the stock solution to the desired concentration with deionized water. The IS solution was prepared by deionized water at the concentration of 200 µg/mL. The standard solutions were stored in brown glass vials to protect from light at 4 °C. Calibration samples were obtained by diluting standard working solutions (10 μL) with drug-free human control serum (90 μL), to span a calibration standard range of 1–100 µg/mL for VCM. QC samples (3, 15, 80 µg/mL) were independently prepared by spiking an appropriate amount of the working standard solution in drug-free human control serum.

### 3.5. Preparation of Human Serum Sample

A simple and rapid sample preparation was applied by protein precipitation in SiroccoTM 96-well filtration plate (Waters Corp., Milford, MA, USA). Standard curve samples (100 μL), QC samples, and subject serum samples were added individually into 96-well plates by an eight-channel hand-held pipettor. To precipitate the serum protein, 200 μL ACN (acetonitrile) solution c ontaining 100 μg/mL of the IS solution was added to each sample (standards, QCs, and samples). Sirocco plates were capped and mixed by vortexing for 3 min and held for 15 min, subsequently placed on a vacuum manifold, and filtered to remove any precipitated material. The filtrate was collected into another 96-well plate and was evaporated to dryness with a nitrogen flow at 37 °C (TURBOVAP 96, Biotage, Uppsala, Sweden). All dry residues were reconstituted by the addition of 50 μL water. Finally, the plates were vortex mixed for 1 min, followed by centrifugation for 10 min at 12,000 rpm at 4 °C using a 5804 R Eppendorf refrigerated centrifuge (Hamburg, Germany). The supernatant was transferred to the autosampler for injection onto the CE.

### 3.6. Liquid Chromatographic Conditions

Two different methods for the determination of PDAP patients and non-PDAP patients were developed separately, and both of them were validated. Separation was performed on a 250 mm × 4.6 mm, 5 μm Waters Symmetry C18 column (Waters Corporation, Milford, MA, USA). Column temperature was 30 °C. The wavelength of the detection was at 236 nm. The mobile phase (a) composed of ACN-20 mM KH_2_PO_4_ buffer (pH 3.0) (7.5:92.5, *v*/*v*) for PDAP patients. The mobile phase (b) composed of ACN-20 mM KH_2_PO_4_ buffer (pH 3.0) (9:91, *v*/*v*) for non-PDAP patients. Both of the flow rates were 1.0 mL/min.

### 3.7. Clinical Study

**Figure 4:**
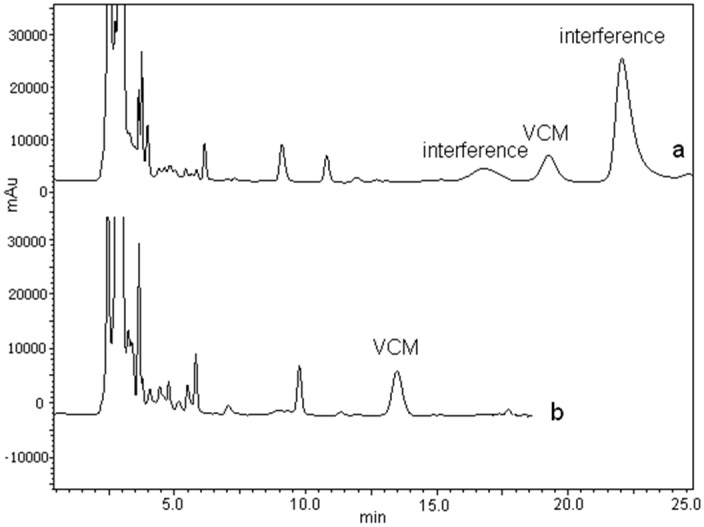
Typical HPLC chromatograms of serum samples from different patients: (**a**) ACN-20 mM KH_2_PO_4_ buffer (pH 3.0) (7.5:92.5, *v*/*v*) for PDAP patients; (**b**) ACN-20 mM KH_2_PO_4_ buffer (pH 3.0) (9:91, *v*/*v*) for non-PDAP patients. Other conditions: Waters Symmetry C18 column (250 mm × 4.6 mm, 5 μm). Column temperature: 30 °C. The wavelength of the detection was 236 nm.

With a BGE based on 25 mM borate buffer with 50 mM sodium SDS and 2% sulfobutyl-β-CD (pH 9.50), the separation of VCM was studied. The method was applied to the analysis of PDAP patients. Due to the combination of hypertension, hyperglycemia, hyperlipidemia, and other diseases, PDAP patients’ medication is complex. The HPLC analysis of PDAP patient’s serum is shown in Figure 4a. A lower percentage of organic solvent was needed to achieve baseline separation, which led to longer analysis time (20 min or more, depending on the medication complexity). In other cases (e.g., endocarditic or meningitis patients), the analysis could be finished in 15 min (Figure 4b), because fewer medicines were used in these patients than those in PDAP patients. Different from HPLC, the separation principle of CD-MEKC is based on the differential partition of the solute among micelle, CD, and aqueous buffer. As Figure 2d shows, no interference was observed during the analysis of the PDAP patients’ serum by CE, and the analysis time was 14 min. In other words, to some extent, compared with the analysis of complex biological samples in HPLC, CD-MEKC may have more advantages.

Patients were treated with intraperitoneal (i.p.) vancomycin (Vancocin, Eli Lilly) for 1.0 g every 5–7 days. Nine PDAP patients’ samples were collected, and the trough concentrations of VCM were measured. No significant changes in renal function were observed before and after treatment. Side effects were not observed in patients who were treated with VCM. All these data are presented in Table 3. The 15–20 µg/mL range was determined in three samples (17.06 µg/mL, 15.39 µg/mL, and 19.25 µg/mL), which was reported as the effective treatment window. Two had VCM levels higher than 20 µg/mL (24.18 µg/mL and 35.22 µg/mL). This may be because most antibiotics have significantly enhanced absorption during peritonitis, and the guideline of ISPD also indicates that i.p. vancomycin is approximately 90% absorbed in the presence of peritonitis. In view of patients’ residual renal function and peritoneal permeability, levels <15 µg/mL were found in four samples (12.88 µg/mL, 5.48 µg/mL, 8.90 µg/mL, and 9.30 µg/mL). ISPD recommends that re-dosing is appropriate once serum vancomycin levels go below 15 µg/mL. As Table 4 shows, CE can been used as an alternative method for VCM concentrations in PDAP patients’ serum.

## 4. Conclusions

Serum-level monitoring of VCM is necessary to ensure adequate therapeutic concentrations while avoiding toxic accumulations in the treatment of PDAP patients. This new CD-MEKC method for the determination of VCM in PDAP patients’ serum described here represents a rapid, sensitive, and efficient analytical method. The analytical characteristics of the proposed method are satisfactory for pharmacokinetic and clinical use. On the other hand, MTX is an important chemotherapeutic agent which was used in this study as IS. The concentration monitoring of this drug is also necessary for reducing incidences of toxicity in cancer patients, especially some children with osteogenesis sarcoma who have to receive high dose chemotherapy. As can be seen in this work, this method can also be developed for the purpose of MTX clinical determination, which is to be a subject in our further work.

## Figures and Tables

**Table 1:** Within- and between-run accuracy and precision in spiked plasma samples (*n* = 5).

Sample Level	LLOQ 1 µg/mL	LQC (Low Quality Control) 3 µg/mL	MQC (Medium Quality Control) 15 µg/mL	HQC (High Quality Control) 80 µg/mL
Within-run accuracy and precision				
Mean ± SD (µg/mL)	1.1732 ± 0.02	3.1029 ± 0.08	14.9600 ± 0.71	81.0559 ± 4.70
Accuracy ± SD (%)	117.07 ± 2.35	103.65 ± 2.74	99.94 ± 4.78	101.53 ± 5.89
RSD (%)	2.00	2.64	4.78	5.80
between-run accuracy and precision				
Mean ± SD (µg/mL)		2.87 ± 0.21	14.50 ± 0.90	80.46 ± 4.43
Accuracy ± SD (%)		95.76 ± 7.07	96.84 ± 6.01	100.79 ± 5.55
RSD (%)		7.38	6.21	5.50

**Table 2:** Recovery values of vancomycin and IS in spiked plasma samples.

		Concentration (µg/mL)	Recovery (%)	RSD (%)
Vancomycin (*n* = 5)	LQC	2.9937 ± 0.0103	84.03 ± 1.88	1.42
	MQC	14.9684 ± 0.1373	91.69 ± 4.67	4.97
	HQC	79.8312 ± 4.43	87.34 ± 1.51	1.55
IS (*n* = 15)		19.5160 ± 0.15	83.67 ± 0.04	0.95

**Table 3:** Stability results of vancomycin in spiked plasma samples (*n* = 5).

	Nominal Concentration (µg/mL)	Measured Concentration (µg/mL) (Mean ± SD)	Accuracy (%)	RSD (%)
Bench-top stability ^a^	LQC	3.271 ± 0.02	107.80%	7.80%
	MQC	15.59 ± 0.09	102.70%	6.20%
	HQC	87.69 ± 0.38	108.40%	5.10%
30-day stability ^b^	LQC	3.18 ± 0.01	104.70%	3.20%
	MQC	15.92 ± 0.08	104.90%	5.80%
	HQC	86.24 ± 0.52	106.60%	0.60%
Autosampler stability ^c^	LQC	3.15 ± 0.01	103.80%	3.10%
	MQC	16.26 ± 0.09	107.10%	6.50%
	HQC	85.57 ± 0.29	105.70%	4.10%
Freeze–thaw stability ^d^	LQC	3.35 ± 0.02	110.40%	7.30%
	MQC	15.88 ± 0.07	104.60%	5.30%
	HQC	88.01 ± 0.23	108.80%	3.80%

^a^: Exposed at ambient temperature (25 °C) for 2 h; ^b^: Stored at −80 °C; ^c^: Kept at ambient temperature (25 °C) for 8 h; ^d^: After three freeze–thaw cycles.

**Table 4:** The concentrations of VCM in PDAP patients’ serum (*n* = 9). CE: capillary electrophoresis.

Concentration Level (µg/mL)	Patient No.	Patients’ Concentration (µg/mL)	
		by CE	by HPLC
>20	1	24.18	25.35
	2	35.22	33.23
15–20	3	17.06	18.50
	4	15.39	16.50
	5	19.25	18.99
<15	6	12.88	14.31
	7	5.48	6.01
	8	8.90	8.07
	9	8.53	9.30
